# Scaling of erythrocyte shape and nucleus size among squamate reptiles: reanalysis points to constrained, proportional rather than adaptive changes

**DOI:** 10.1098/rsos.221513

**Published:** 2023-04-26

**Authors:** Stanisław Bury, Lukáš Kratochvíl, Zuzana Starostová

**Affiliations:** ^1^ Department of Comparative Anatomy, Institute of Zoology and Biomedical Research, Jagiellonian University, Gronostajowa 9, 30-387 Kraków, Poland; ^2^ Department of Ecology, Faculty of Science, Charles University, Viničná 7, 12844 Prague, Czech Republic; ^3^ Department of Zoology, Faculty of Science, Charles University, Viničná 7, 12844 Prague, Czech Republic

**Keywords:** cell size, cell shape, N : C ratio, erythrocytes, scaling, reptiles

## Abstract

Small erythrocytes might be beneficial for blood rheology, as they contribute less to blood viscosity than large erythrocytes. We predicted that rheological disadvantages of larger erythrocytes could be alleviated by relatively smaller nucleus size in larger cells allowing higher flexibility and by more elongated shape. Across squamate reptiles, we found that species with larger erythrocytes tend to have smaller ratio of nucleus size to cell size (N : C ratio), but that larger erythrocytes tend to be rounder, not more elongated. Nevertheless, we document that in fact nucleus area changes with erythrocyte area more or less linearly, which is also true for the relationship between cell length and cell width. These linear relationships suggest that nucleus size and cell size, and cell width and cell length, might be constrained to largely proportional mutual changes. The shifts in widely used N : C ratio and elongation ratio (cell length/cell width) with cell size might be misleading, as they do not reflect adaptive or maladaptive changes of erythrocytes, but rather mathematically trivial scaling of the ratios of two variables with a linear relationship with non-zero intercepts. We warn that ratio scaling without analyses of underlying patterns of evolutionary changes can lead to misinterpretation of evolutionary processes.

## 1. Introduction

Cells that build up organisms express a considerable intra- and inter-specific variation in size, which can affect energy budget and life history [1–3]. Organisms composed of many smaller cells have a larger total surface of cellular membranes compared with organisms of the same size, but with larger cells [2,4]. All else being equal, a higher amount of membranes would enhance intercellular connectivity and transportation, a prerequisite for a high metabolic rate [5]. Simultaneously, we should also expect higher energy expenditure for membrane maintenance. The role of transmembrane processes in shaping metabolic rates should be particularly important for erythrocytes, cells specialized for gas transport. High oxygen demands can be secured through more efficient oxygen uptake and release, due to larger (relative to volume) membrane surface in smaller erythrocytes. Empirical data indicate a correlation between erythrocyte size and energy expenditure. For example, interspecific variation in standard metabolic rate in geckos and birds is inversely related to erythrocyte size, and snakes maintained at a higher temperature having higher metabolic rate tend to express smaller erythrocytes [6–8]. The higher efficiency of small erythrocyte size for oxygen uptake and release was demonstrated *in vitro* as well [9,10].

The size, but also the shape of erythrocytes is important for blood circulation. Fluid viscosity increases with size of circulating particles [11] and the transport of smaller cells through vessels is thus easier [12]. Conceivably, under equal erythrocyte count, the size of erythrocytes correlates with blood density and viscosity [13]. In turn, more elongated erythrocytes can move more easily through vessels [14,15]. High blood viscosity impedes blood circulation and thus elevates the workload of the cardiovascular system. Therefore, the metabolic costs of maintaining optimal blood flow can be paradoxically elevated when erythrocytes are large, despite larger cells being expected to be associated with lower energy expenses. Such effects could be particularly pronounced in nucleated erythrocytes, because a rigid nucleus is thought to reduce cell deformability that would allow the cell to adjust to vessel diameter [12]. Nucleus size is generally positively correlated with genome size; however, the relationship is quite loose and can be to a certain degree disentangled [16]. We focus here on the relationship between cell size and nucleus size, not genome size, as we assume that nucleus size is more important for cell deformability. Constraints for cell deformation can be an important factor driving the major evolutionary changes in erythrocyte morphology. Erythrocyte size considerably varies among vertebrates [17,18]. Specifically, the size of erythrocytes is significantly smaller in birds compared with non-avian reptiles [19], while mammals have even smaller and entirely enucleated erythrocytes, which facilitate changes in cell shape to adjust to small capillary diameters and high blood flow [12]. Prevalent erythrocyte enucleation has also been observed in plethodontid salamanders with miniaturized or attenuated bodies and large cells and, similarly as in mammals, it is hypothesized that enucleation helps to ease blood flow [20].

Non-avian reptiles are characterized by large nucleated erythrocytes. Furthermore, they possess almost half as low density of capillaries as mammals [21]. At the same time, the elongated bodies in many squamates and other reptiles necessitate longer vessels relative to body mass. The mentioned factors are known to oppose blood flow; however, these constraints to blood circulation can be alleviated by adjustments of erythrocyte morphology. Specifically, we predict that species with larger erythrocytes should have relatively smaller size of nucleus to permit greater deformability (Hypothesis I), which should be manifested by a negative relationship between N : C ratio (nucleus size/cell size) and cell size. An adaptive pattern should be underlaid by a disproportionately smaller increase in nucleus size with cell size in erythrocytes. Second, we expect that larger cells should have more elongated shape so that they can flow more easily through capillaries (Hypothesis II; [14]), i.e. that elongation (measured as the cell length/cell width ratio) should be positively correlated with cell size. The adaptive evolutionary change in cell shape would imply that changes in cell length should increase relative to changes in cell width with increasing cell size. We tested these predictions about evolutionary changes within a comparative phylogenetic framework on a large dataset in squamate reptiles. However, since using ratios can lead to misleading results and interpretations (e.g. [22–25]), we also applied an alternative, allometric approach allowing deeper insights into processes forming the scaling pattern.

## 2. Methods

### 2.1. Data collection

Data on the erythrocytes in squamate reptiles were extracted from published sources. We collected data on four variables describing erythrocyte morphology obtained from measurements on blood smears: cell and nucleus sizes expressed as area, cell length and cell width. If the data on nucleus or cell area were not provided, but instead lengths and widths were available, we calculated the area using the formula for ellipse area *p* = π ∗ *a* ∗ *b,* where *p* is ellipse area, *a* is cell width/2 and *b* is cell length/2.

Where multiple records were found for a given species we averaged them to get a single value. In total, we collected data on 181 species of squamates (84 snakes and 97 species of lizards including amphisbaenians). We excluded from the dataset several entries where we had at least three different values and sources for one species and observed an obvious deviation in one value. In such rare cases, we excluded from the dataset also other species/values included in the same publication (see electronic supplementary material for details). Outliers from the relationship between cell length and width and between area of cells and their nuclei identified as species with an absolute value of standardized residual larger than three [26] were also removed from the datasets (four species in total).

### 2.2. Data analysis

Phylogenetic correction is required in comparative studies to account for the non-independence of species data that share a common evolutionary history. When comparing traits among different species, it is important to consider the relatedness between them, as species that are closely related are more likely to share similar characteristics than those that are distantly related. Phylogenetic correction allows us to control for the effects of evolutionary history on our analyses, by incorporating information about the evolutionary relationships between species into our statistical models. This approach can improve the accuracy and power of our comparative analyses, and help us to better understand the underlying drivers of trait variation and evolution. Therefore, we analysed the data using phylogenetic least-squares (PGLS) regression taking into account the reconstructed phylogeny of squamates [27]. We employed the ‘phylolm’ function in the phylolm package in R [28] with a lambda model of phylogenetic covariance [29,30]. Pagel's lambda (*λ*; [31]) is used to measure the strength of the phylogenetic signal and typically varies between 0, indicating no effect of phylogenetic signal, and 1, corresponding to a strong effect of phylogeny. To test for the relationship between cell size and relative nucleus size (Hypothesis I), we first calculated N : C ratio by dividing nucleus area by the erythrocyte area. Subsequently, we explored whether variables used to calculate the N : C ratio express a linear or nonlinear relationship. To do this, we ran the null model (intercept only) and two additional models that included either cell size as the only predictor or cell size and its quadratic term. The fits of the PGLS models were evaluated by the Akaike information criterion (AIC; [32]). We selected the best model as the model with the lowest AIC. Models with the same number of parameters and differences in the AIC (ΔAIC) < 2 were taken as equivalent. Among models with ΔAIC < 2, we preferred the one with the smallest number of parameters. The same approach was applied to test for the Hypothesis II. After testing for the relationship between cell shape, calculated as length-to-width ratio, and erythrocyte area (Hypothesis II), we tested whether the relationship between cell length and cell width is best explained by a linear model or a model that included quadratic cell width and compared AIC of these models.

## 3. Results and discussion

Erythrocyte area of squamates included in our study ranged from 73 µm^2^ in snake-eyed lizard (*Ophisops elegans*) to 259 µm^2^ in Komodo dragon (*Varanus komodoensis*). The nucleus area varied from 9.20 µm^2^ in African rock python (*Python sebae*) to 38 µm^2^ in common agama (*Agama agama*).

**Figure 1.**
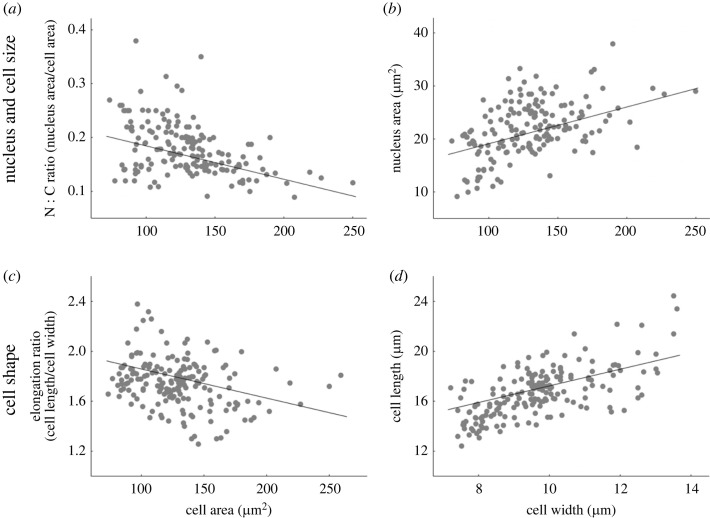
Relationships between morphological variables of erythrocytes. (*a*) N : C ratio (nucleus size/cell size) and (*c*) elongation ratio (cell length/width) are negatively correlated to cell size expressed as area. However, the relationship that built up these ratio traits is well approximated by a linear function indicating proportional changes of nucleus size with changes in cell size (*b*), and proportional changes in cell length with changes in cell width (*d*). Regression lines indicate a significant relationship corrected for the phylogeny.

Using the traditional approach based on ratios, we found statistically significant support for the negative relationship between N : C ratio and cell size (figure 1*a*, table 1), showing that larger erythrocytes have relatively smaller nuclei. We also detected a significant, but contrary to the prediction, negative association between the elongation ratio and erythrocyte size (figure 1*c*, table 2), which indicates that larger cells tend to be rounder. From the rheological perspective, these significant relationships could be interpreted as a support for the adaptive adjustment of the relative nucleus size, but maladaptive adjustment in cell shape to changes in cell size [14]. However, these interpretations are not supported by the analyses based on absolute values, not ratios. Specifically, the relationships between nucleus area and cell area as well as between cell length and cell width were best explained by the linear function (figure 1*b,d*, tables 1 and 2). The linearity implies that changes of nucleus size and changes of cell size, and changes in cell length and changes in cell width, respectively, are largely proportional. The proportional changes are not expected in the context of adaptive changes in nuclear size and cell shape. The generally proportional changes could point to a constrained relationship in changes between the studied variables, although variability around this relationship can be found.

**Table 1.** Results of phylogenetically corrected models testing for the relationships between N : C ratio (nucleus size/cell size) and red blood cell size (area) as well as between variables underlying N : C ratio. When more models were tested, the best model is in italics.

model	AIC	ΔAIC	lambda (*λ*)	*R*^2^		estimate	s.e.	*p*-value
1. nucleus size and cell size								
1a. relative nucleus size (N : C_ratio_; nucleus_area_/cell_area_) and cell size (cell_area_)								
N : C_ratio_ ∼ cell_area_	−625.0	NA	0.605	0.155	intercept	0.246	0.018	≪0.001
					cell_area_	−0.6 * 10^−3^	0.1 * 10^−3^	≪0.001
1b. absolute nucleus size (nucleus_area_) and cell size (cell_area_)								
${\mathrm{nucleus}}_{\mathrm{area}}∼{\mathrm{cell}}_{\mathrm{area}}+{\mathrm{cell}}_{\mathrm{area}}^{2}$	966.2	0	0.549	0.153	intercept	2.891	5.611	0.607
					cell_area_	0.203	0.076	0.008
					${\mathrm{cell}}_{\mathrm{area}}^{2}$	−0.4 * 10^−3^	−0.2 * 10^−3^	0.075
* nucleus*_*area*_ ∼ *cell*_*area*_	*967.5*	*1.3*	*0.573*	*0.136*	intercept	12.171	2.175	≪0.001
					cell_area_	0.069	0.014	≪0.001
nucleus_area_ ∼ 1 (the null model)	989.8	23.6	0.596	0	intercept	20.849	1.487	≪0.001

**Table 2.** Results of phylogenetically corrected models testing for the relationships between elongation ratio (cell length/width) and red blood cell size (area) as well as between variables underlying elongation ratio. When more models were tested, the best model is in italics.

model	AIC	ΔAIC	lambda (*λ*)	*R*^2^		estimate	s.e.	*p*-value
2. cell shape and size								
2a. cell shape (elongation_ratio_; cell_length_/cell_width_) and cell size (cell_area_)								
elongation_ratio_ ∼ cell_area_	−106.0	NA	0.371	0.117	intercept	2.092	0.074	≪0.001
					cell_area_	−0.2 * 10^−2^	0.4 * 10^−3^	≪0.001
2b. cell_length_ and cell_width_								
${\mathrm{cell}}_{\mathrm{length}}∼{\mathrm{cell}}_{\mathrm{width}}+{\mathrm{cell}}_{\mathrm{width}}^{2}$	611.1	0	0.745	0.30	intercept	17.46	3.892	≪0.001
					cell_width_	−0.698	0.760	0.359
					${\mathrm{cell}}_{\mathrm{width}}^{2}$	0.066	0.037	0.075
*cell_length_* ∼ *cell_width_*	*612.1*	*1*	*0.684*	*0.293*	intercept	10.570	0.873	≪0.001
					cell_width_	0.666	0.078	≪0.001
cell_length_ ∼ 1 (the null model)	671.3	60.2	0.773	0	intercept	16.873	0.631	≪0.001

**Figure 2.**
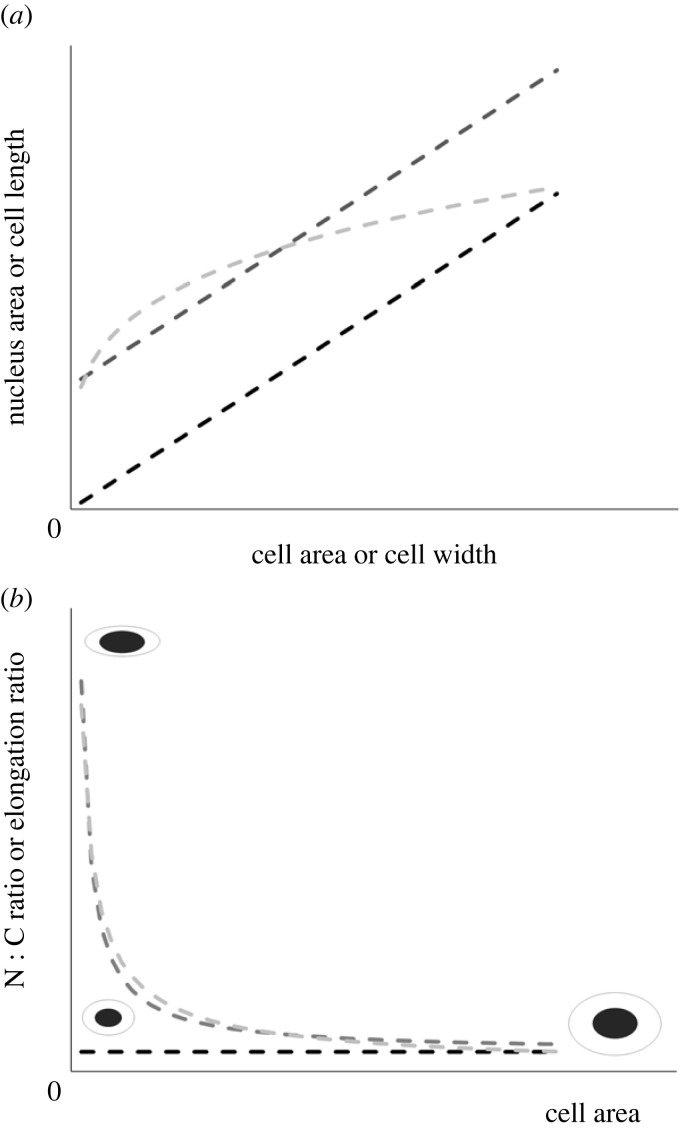
Schematic depiction of potential relationships between variables (nucleus and cell area, or cell length and width) used for the construction of N : C (nucleus size/cell size) and elongation (cell length/width) ratios. Linear relationships between numerator and denominator with a zero intercept (graph (*a*), black line—proportionality) result in a constant ratio (graph (*b*)), while linear function with exactly the same slope but a positive intercept results in a negative nonlinear scaling of ratios (graphs (*a*) and (*b*), dark grey lines) statistically indistinguishable from the ratio scaling derived from a nonlinear relationship between numerator and denominator (graphs (*a*) and (*b*), light grey lines; power function is shown here as an example of a nonlinear function).

Importantly, in both N : C ratio and elongation ratio, the corresponding linear relationships between the variables in the numerators (nucleus size and erythrocyte length) and the denominators (erythrocyte size and erythrocyte width) have non-zero positive intercepts. Non-zero positive intercepts strongly affect variation in ratios and consequently result in a negative scaling of ratios, which is statistically impossible to differentiate from a pattern originating from a nonlinear relationship between a numerator and a denominator ([24]; figure 2). The patterns based on ratios are hence difficult to interpret and can obscure the underlying relationships between variables. As shown here for squamates, the negative scaling of the N : C ratio with cell size might be attributed to linear scaling of nucleus size with cell size, i.e. proportional changes of these characteristics. However, we stress that the increase of nucleus-to-cell size in squamates is not proportional, but only linear. The proportional function *y* = *a* ∗ *x* is a special case of a linear function *y* = *a* ∗ *x* + *b*; the changes Δ*x* and Δ*y* are proportional in every linear relationship, but the ratio *y*/*x* is a function of *b*/*y* and hence constant only for zero intercept, i.e. for *b* = 0. When *b* is different from zero, the ratio necessarily scales with *y*, even when the changes between *x* and *y* are proportional, i.e. when Δ*x*/Δ*y* is constant.

The use of ratios is common in various fields of biology, because they are easy to calculate, and ratios are believed to standardize for the variation in the denominator variable and/or for detecting the scaling pattern between variables [22,33]. The validity of the naive use of ratios was questioned many times [23,25,33], particularly because the variation in ratios can be strongly biased by the type of mathematical relationship between numerators and denominators [24]. Our results indicate that methodological concerns can also be raised in the field of erythrocyte morphology, which has become of growing interest over the last two decades (e.g. [2,4,15,34,35]). Many studies analysing N : C and elongation ratio did not evaluate the relationships between traits used for the derivation of the ratio [15,34,36–41]. That also includes a recent study that tested the relationship between cell size and relative nucleus size, similarly as in our research, but on a larger representation of vertebrates, but also invertebrates, plants and bacterial taxa [42]. Both the present study and Malerba & Marshall [42] found a negative association between N : C ratio and cell size, but the underlying pattern of evolutionary changes producing the shift in the N : C ratio with cell size was tested only here. As we demonstrated, the negative correlation between N : C ratio and cell size can emerge from a simple linear relationship between numerator and denominator, which has very different evolutionary interpretations compared with the nonlinearity (assuming non-proportional changes between nucleus size and cell size). We therefore call for attention to evolutionary and functional interpretations based on this ratio used for over 90 years [43].

Are erythrocytes adapted to easier circulation, even when we did not find convincing support for either of our hypotheses on their morphology? Erythrocytes express considerable phenotypic plasticity and flexibility in size in response to energy requirements [8,44,45]. Such dynamic size variation might attenuate constraints imposed by shape or nucleus [8], but was not covered in our study. We suggest that alternative mechanisms beyond morphological changes to attenuate the proposed limitations of large erythrocytes should be considered as well. Besides size and morphology, erythrocyte deformability can be influenced by other cellular factors such as cell membrane and cytoskeletal properties or intracellular viscosity [46,47]. Other factors affecting blood rheology and viscosity may include adjustments in haematological traits such as erythrocyte number and haematocrit [8,19,48] or changes in blood parameters such as osmolality or plasma viscosity [12,49,50]. Another limitation of our study is that we are in reptiles missing three-dimensional data on cell and nucleus size and shape, and we are still limited to two-dimensional data from blood smears. Relevant for future studies can also be differences in the morphology of vessels, particularly capillaries [51,52]. Data on capillary diameters in various species of reptiles are generally lacking, but could provide additional insight into evolutionary changes concerning blood rheology and thus help to understand the effects of erythrocyte morphology on circulation.

In summary, our study applied an explicit allometric approach to investigate the mutual evolutionary changes between cell width and cell length, and between nucleus size and cell size, in squamate reptiles. This approach helped us to test if there is a linear or nonlinear relationship between studied traits, which is often practically indistinguishable when a more traditional method based on ratios is applied (figure 2). Importantly, we show that the inference based on analyses involving only ratios can be incomplete or misleading. Our results indicate that the changes in nucleus size and cell size, and in cell width and cell length were largely proportional, which contrasts with previous studies based on ratios that assumed disproportionately smaller changes in nucleus size compared with cell size with cell size increase. However, further functional studies are needed to determine whether erythrocyte changes with size affect other characteristics besides morphology allowing to alleviate potential negative effects on circulation connected with larger erythrocyte size.

## Data Availability

The data are provided in the electronic supplementary material [53].

## References

[1] Szarski H. 1983 Cell-size and the concept of wasteful and frugal evolutionary strategies. J. Theor. Biol. **105**, 201-209. (10.1016/S0022-5193(83)80002-2) 6656279

[2] Kozłowski J, Konarzewski M, Gawelczyk T. 2003 Cell size as a link between noncoding DNA and metabolic rate scaling. Proc. Natl Acad. Sci. USA **100**, 14 080-14 085. (10.1073/pnas.2334605100) PMC283549 14615584

[3] Kozłowski J, Czarnoleski M, François-Krassowska A, Maciak S, Pis T. 2010 Cell size is positively correlated between different tissues in passerine birds and amphibians, but not necessarily in mammals. Biol. Lett. **6**, 792-796. (10.1098/rsbl.2010.0288) 20462886 PMC3001361

[4] Czarnoleski M, Labecka AM, Starostová Z, Sikorska A, Bonda-Ostaszewska E, Woch K, Kubička L, Kratochvíl L, Kozlowski J. 2017 Not all cells are equal: temperature and sex effects on the size of different cell types in the Madagascar ground gecko *Paroedura picta*. Biol. Open **6**, 1149-1154. (10.1242/bio.025817) 28630354 PMC5576080

[5] Antoł A, Labecka AM, Horváthová T, Sikorska A, Szabla N, Bauchinger U, Kozlowski J, Czarnoleski M. 2020 Effects of thermal and oxygen conditions during development on cell size in the common rough woodlice *Porcellio scaber*. Ecol. Evol. **10**, 9552-9566. (10.1002/ece3.6683) 32953083 PMC7487255

[6] Gregory TR. 2002 A bird's-eye view of the C-value enigma: genome size, cell size, and metabolic rate in the class Aves. Evolution **56**, 121-130. (10.1111/j.0014-3820.2002.tb00854.x) 11913657

[7] Starostová Z, Kubička L, Kozlowski J, Konarzewski M, Kratochvíl L. 2009 Cell size but not genome size affects scaling of metabolic rate in eyelid geckos. Am. Nat. **174**, E100-E105. (10.1086/603610) 19604072

[8] Bury S, Bury A, Sadowska ET, Cichon M, Bauchinger U. 2019 More than just the numbers—contrasting response of snake erythrocytes to thermal acclimation. Sci. Nat. **106**, 24. (10.1007/s00114-019-1617-x) 31069520

[9] Holland RAB, Forster RE. 1966 The effect of size of red cells on the kinetics of their oxygen uptake. J. Gen. Physiol. **49**, 727-742. (10.1085/jgp.49.4.727) 5943611 PMC2195500

[10] Yamaguchi K, Jürgens KD, Bartels H, Piiper J. 1987 Oxygen transfer properties and dimensions of red blood cells in high-altitude camelids, dromedary camel and goat. J. Comp. Physiol. B **157**, 1-9. (10.1007/BF00702722) 3571563

[11] Nguyen CT, Desgranges F, Roy G, Galanis N, Maré T, Boucher S, Mintsa HA. 2007 Temperature and particle-size dependent viscosity data for water-based nanofluids – hysteresis phenomenon. Int. J. Heat Fluid. Fl. **28**, 1492-1506. (10.1016/j.ijheatfluidflow.2007.02.004)

[12] Windberger U, Baskurt OK. 2007 Comparative hemorheology. In Handbook of hemorheology and hemodynamics (eds OK Baskurt, MR Hardeman, MW Rampling, HJ Meiselman), pp. 267-284. Amsterdam, The Netherlands: IOS Press.

[13] Snyder GK, Sears RD. 2006 Red blood cell size and the Fåhraeus–Lindqvist effect. Can. J. Zool. **84**, 419-424. (10.1139/z06-011)

[14] Chien S, Usami S, Dellenback RJ, Bryant CA. 1971 Comparative hemorheology – hematological implications of species differences in blood viscosity. Biorheology **8**, 35-57. (10.3233/BIR-1971-8106) 4103884

[15] Penman Z, Deeming DC, Soulsbury CD. 2022 Ecological and life-history correlates of erythrocyte size and shape in Lepidosauria. J. Evol. Biol. **35**, 708-718. (10.1111/jeb.14004) 35384114 PMC9322653

[16] Starostová Z, Kratochvíl L, Flajšhans M. 2008 Cell size does not always correspond to genome size: phylogenetic analysis of genome size in eublepharid geckos. Zoology **111**, 377-384. (10.1016/j.zool.2007.10.005) 18595679

[17] Gulliver G. 1875 Observations on the sizes and shapes of the red corpuscles of the blood of vertebrates, with drawings of them to a uniform scale, and extended and revised tables of measurements. Proc. Zool. Soc. Lond. **1875**, 474-495.

[18] Snyder GK, Sheafor BA. 1999 Red blood cells: centerpiece in the evolution of the vertebrate circulatory system. Am. Zool. **39**, 189-198. (10.1093/icb/39.2.189)

[19] Hawkey CM, Bennett PM, Gascoyne SC, Hart MG, Kirkwood JK. 1991 Erythrocyte size, number and haemoglobin content in vertebrates. Br. J. Haematol. **77**, 392-397. (10.1111/j.1365-2141.1991.tb08590.x) 2012765

[20] Mueller RL, Gregory TR, Gregory SM, Hsieh A, Boore JL. 2008 Genome size, cell size, and the evolution of enucleated erythrocytes in attenuate salamanders. Zoology **111**, 218-230. (10.1016/j.zool.2007.07.010) 18328681 PMC2435017

[21] Pough FH. 1980 Blood oxygen transport and delivery in reptiles. Am. Zool. **20**, 173-185. (10.1093/icb/20.1.173)

[22] Kronmal RA. 1993 Spurious correlation and the fallacy of the ratio standard revisited. J. R. Stat. Soc. Ser. A **156**, 379-392. (10.2307/2983064)

[23] Jasieński M, Bazzaz FA. 1999 The fallacy of ratios and the testability of models in biology. Oikos **84**, 321-326. (10.2307/3546729)

[24] Kratochvíl L, Rovatsos M. 2022 Ratios can be misleading for detecting selection. Curr. Biol. **32**, R28-R30. (10.1016/j.cub.2021.11.066) 35015989

[25] Lolli L, Batterham AM, Kratochvíl L, Flegr J, Weston KL, Atkinson G. 2017 A comprehensive allometric analysis of 2nd digit length to 4th digit length in humans. Proc. R. Soc. B **284**, 20170356. 10.1098/rspb.2017.0356 PMC5489719 28659446

[26] Bollen KA, Jackman RW. 1990 Regression diagnostics: an expository treatment of outliers and influential cases. In Modern methods of data analysis (eds J Fox, LJ Scott), pp. 257-291. Newbury Park, CA: Sage.

[27] Zheng Y, Wiens JJ. 2016 Combining phylogenomic and supermatrix approaches, and a time-calibrated phylogeny for squamate reptiles (lizards and snakes) based on 52 genes and 4162 species. Mol. Phylogenet. Evol. **94**, 537-547. (10.1016/j.ympev.2015.10.009) 26475614

[28] Ho LST, Ane C. 2014 A linear-time algorithm for Gaussian and non-Gaussian trait evolution models. *Syst. Biol.* **63**, 397-408. (10.1093/sysbio/syu005) 24500037

[29] Grafen A. 1989 The phylogenetic regression. Phil. Trans. R. Soc. lond. B **326**, 119-157. (10.1098/rstb.1989.0106) 2575770

[30] Freckleton RP, Harvey PH, Pagel M. 2002 Phylogenetic analysis and comparative data: a test and review of evidence. Am. Nat. **160**, 712-726. (10.1086/343873) 18707460

[31] Pagel M. 1999 Inferring the historical patterns of biological evolution. Nature **401**, 877-884. (10.1038/44766) 10553904

[32] Akaike H. 1973 Information theory and an extension of the maximum likelihood principle. In Proc. of the 2nd Int. Symp. on Information Theory, 2–8 September 1971 (eds BN Petrovand, S Caski), pp. 267-281. Budapest, Hungary: Akademiai Kaido.

[33] Beaupre SJ. 2005 Ratio representations of specific dynamic action (mass-specific SDA and SDA coefficient) do not standardize for body mass and meal size. Physiol. Biochem. Zool. **78**, 126-131. (10.1086/425195) 15702471

[34] Soulsbury CD, Dobson J, Deeming CD, Minias P. 2021 Energetic lifestyle drives size and shape of avian erythrocytes. Integr. Comp. Biol. **62**, 71-80. (10.1093/icb/icab195) PMC9375138 34581789

[35] Janiga M, Haas M, Kufelová M. 2017 Age, sex and seasonal variation in the shape and size of erythrocytes of the alpine accentor, *Prunella collaris* (Passeriformes: Prunellidae). Eur. Zool. J. **84**, 583-590. (10.1080/24750263.2017.1403656)

[36] Gregory TR. 2001 The bigger the C-value, the larger the cell: genome size and red blood cell size in vertebrates. Blood Cells Mol. Dis. **27**, 830-843. (10.1006/bcmd.2001.0457) 11783946

[37] Chen B, Co C, Ho CC. 2015 Cell shape dependent regulation of nuclear morphology. Biomaterials **67**, 129-136. (10.1016/j.biomaterials.2015.07.017) 26210179 PMC4626019

[38] Jung J, Matemba LE, Lee K, Kazyoba PE, Yoon J, Massaga JJ, Kim K, Kim DJ, Park Y. 2016 Optical characterization of red blood cells from individuals with sickle cell trait and disease in Tanzania using quantitative phase imaging. Sci. Rep. **6**, 31698. (10.1038/srep31698) 27546097 PMC4992839

[39] McKinley KL, Stuurman N, Royer LA, Schartner C, Castillo-Azofeifa D, Delling M, Klein OD, Vale RD. 2018 Cellular aspect ratio and cell division mechanics underlie the patterning of cell progeny in diverse mammalian epithelia. eLife **7**, e36739. (10.7554/eLife.36739) 29897330 PMC6023609

[40] Moore MJ, Sebastian JA, Kolios MC. 2019 Determination of cell nucleus-to-cytoplasmic ratio using imaging flow cytometry and a combined ultrasound and photoacoustic technique: a comparison study. J. Biomed. Opt. **24**, 106502. (10.1117/1.JBO.24.10.106502) 31625322 PMC7000884

[41] Grant NA, Magid AA, Franklin J, Dufour Y, Lenski RE. 2020 Changes in cell size and shape during 50,000 generations of experimental evolution with *Escherichia coli*. J. Bacteriol. **203**, e00469-20. (10.1128/JB.00469-20) PMC8088598 33649147

[42] Malerba ME, Marshall DJ. 2021 Larger cells have relatively smaller nuclei across the Tree of Life. Evol. Lett. **5**, 306-314. (10.1002/evl3.243) 34367657 PMC8327945

[43] Sinnott EW, Trombetta VV. 1936 The cytonuclear ratio in plant cells. Am. J. Bot. **23**, 602-606. (10.1002/j.1537-2197.1936.tb09032.x)

[44] Goodman RM, Heah TP. 2010 Temperature-induced plasticity at cellular and organismal levels in the lizard *Anolis carolinensis*. Integr. Zool. **5**, 208-217. (10.1111/j.1749-4877.2010.00206.x) 21392339

[45] Hermaniuk A, Rybacki M, Taylor JR. 2016 Low temperature and polyploidy result in larger cell and body size in an ectothermic vertebrate. Physiol. Biochem. Zool. **89**, 118-129. (10.1086/684974) 27082722

[46] Meiselman HJ. 1981 Morphological determinants of red cell deformability. Scand. J. Clin. Lab. Investig. **41**(Suppl. 156), 27-34. (10.3109/00365518109097426) 6948391

[47] Huisjes R, Bogdanova A, van Solinge WW, Schiffelers RM, Kaestner L, van Wijk R. 2018 Squeezing for life – properties of red blood cell deformability. Front. Physiol. **9**, 656. (10.3389/fphys.2018.00656) 29910743 PMC5992676

[48] Nemeth N, Alexy T, Furka A, Baskurt OK, Meiselman HJ, Furka I, Miko I. 2009 Inter-species differences in hematocrit to blood viscosity ratio. Biorheology **46**, 155-165. (10.3233/BIR-2009-0533) 19458418

[49] Nader E et al. 2019 Blood rheology: key parameters, impact on blood flow, role in sickle cell disease and effects of exercise. Front. Physiol. **10**, 1329. (10.3389/fphys.2019.01329) 31749708 PMC6842957

[50] Varga A, Matrai AA, Barath B, Deak A, Horvath L, Nemeth N. 2022 Interspecies diversity of osmotic gradient deformability of red blood cells in human and seven vertebrate animal species. Cells **11**, 1351. (10.3390/cells11081351) 35456029 PMC9026962

[51] Wallach V. 1998 Pulmonary system: the lung of snakes. In Biology of the reptilia. Volume 19. Morphology G. Visceral organs (eds C Gans, S Abbot), pp. 93-295. New York, NY: SSAR.

[52] Sheehy III CM, Albert JS, Lillywhite HB. 2016 The evolution of tail length in snakes associated with different gravitational environments. Funct. Ecol. **30**, 244-254. (10.1111/1365-2435.12472)

[53] Bury S, Kratochvíl L, Starostová Z. 2023 Scaling of erythrocyte shape and nucleus size among squamate reptiles: reanalysis points to constrained, proportional rather than adaptive changes. Figshare. (10.6084/m9.figshare.c.6602821) PMC10130710 37122952

